# Don’t Crowd (Poetical)

**Published:** 1875-01

**Authors:** 


[There is much of excellent ethics in the following, from the pen of Charles Dickens.—Editor.]

*DON'T CROWD.*

Don't crowd—the world is large enough
For you as well as me;
The doors of all are open wide—
The realm of thought is free.
In all earth's places you are right
To chase the best you can,
Provided that you do not try
To crowd some other man.
Don't crowd the good from out your heart
By fostering all that's bad;
But give to every virtue room—
The best that may be had:
To each day's record such a one
That you may well be proud;
Give each his right—give each his room,
And never try to crowd.

